# Two-dimensional estimates of left ventricular strains are significantly affected by through-plane motion

**DOI:** 10.1186/1532-429X-16-S1-O39

**Published:** 2014-01-16

**Authors:** Jonathan D Suever, Gregory J Wehner, Linyuan Jing, David Powell, Christopher M Haggerty, Xiaodong Zhong, Frederick H Epstein, Brandon K Fornwalt

**Affiliations:** 1 Pediatrics, University of Kentucky, Lexington, Kentucky, USA; 2 Biomedical Engineering, University of Kentucky, Lexington, Kentucky, USA; 3 MR R&D Collaborations, Siemens Healthcare, Atlanta, Georgia, USA; 4 Department of Biomedical Engineering, University of Virginia', Charlottesville, Virginia, USA

## Background

Advanced measures of cardiac mechanics such as left ventricular (LV) strains can be used in conjunction with classical biomarkers to gauge cardiovascular health and improve prediction of patient outcomes. Several imaging techniques, including displacement-encoded magnetic resonance imaging (DENSE), are used to non-invasively assess cardiac mechanics. These data are predominantly acquired in two dimensions (2D) due to simplified post-processing and shorter acquisition times; however, this type of acquisition and subsequent analysis cannot account for through-plane motion caused by longitudinal contraction of the left ventricle. We hypothesized that through-plane motion has a significant effect on 2D strain estimates.

## Methods

Cine DENSE data were acquired in eight healthy volunteers (Age: 27 ± 3 years) with a 3T Siemens Tim Trio scanner. Short-axis slices with 2.8 mm in-plane resolution and an 8 mm slice thickness were acquired to span the entire LV. Displacements were encoded in both through-plane and in-plane directions with an effective temporal resolution of 34 ms. Endocardial and epicardial boundaries were delineated on the magnitude image of all short axis DENSE images. Radial and circumferential strains were computed based upon the deformation of the myocardium relative to the end-diastolic frame. Through-plane displacements were ignored for 2D analysis. For three-dimensional (3D) analysis, a 3D representation of the myocardium derived from the same endocardial and epicardial boundaries was deformed using the measured displacement field. The resulting radial and circumferential strain values were compared directly between the 2D and 3D analyses using a two-tailed paired t-test.

## Results

Two dimensional processing consistently overestimated radial strain and underestimated circumferential strain. Peak circumferential strain was significantly different at the basal and mid-ventricular segments (p = 0.001 and 0.009, respectively). Peak radial strain decreased from the base to the apex in both 2D and 3D analyses; however, 2D significantly overestimated radial strain at the mid-ventricular and apical slices compared to 3D (p = 0.002). Global peak radial and circumferential strains from 3D were 30 ± 5% and -20 ± 2%, respectively, compared to 36 ± 5% and -18 ± 2% for 2D (both p < 0.001).

## Conclusions

Two-dimensional imaging methods for assessing left ventricular mechanics consistently overestimate radial strain and underestimate circumferential strain when compared to three-dimensional imaging. This limitation of two-dimensional imaging is likely due to the through-plane motion of the heart, which is ignored in two-dimensional techniques but easily accounted for when using three-dimensional techniques. Future research needs to determine the clinical and prognostic significance of this difference.

## Funding

This research was funded in part by an NIH Early Independence Award to BKF (DP5 OD012132); contributions made by local businesses and individuals through a partnership between Kentucky Children's Hospital and Children's Miracle network; and the University of Kentucky Cardiovascular Research Center, grant UL1RR033173 from the National Center for Research Resources (NCRR), funded by the Office of the Director, National Institutes of Health (NIH) and supported by the NIH Roadmap for Medical Research. The content is solely the responsibility of the authors and does not necessarily represent the official views of the funding sources.

**Figure 1:**
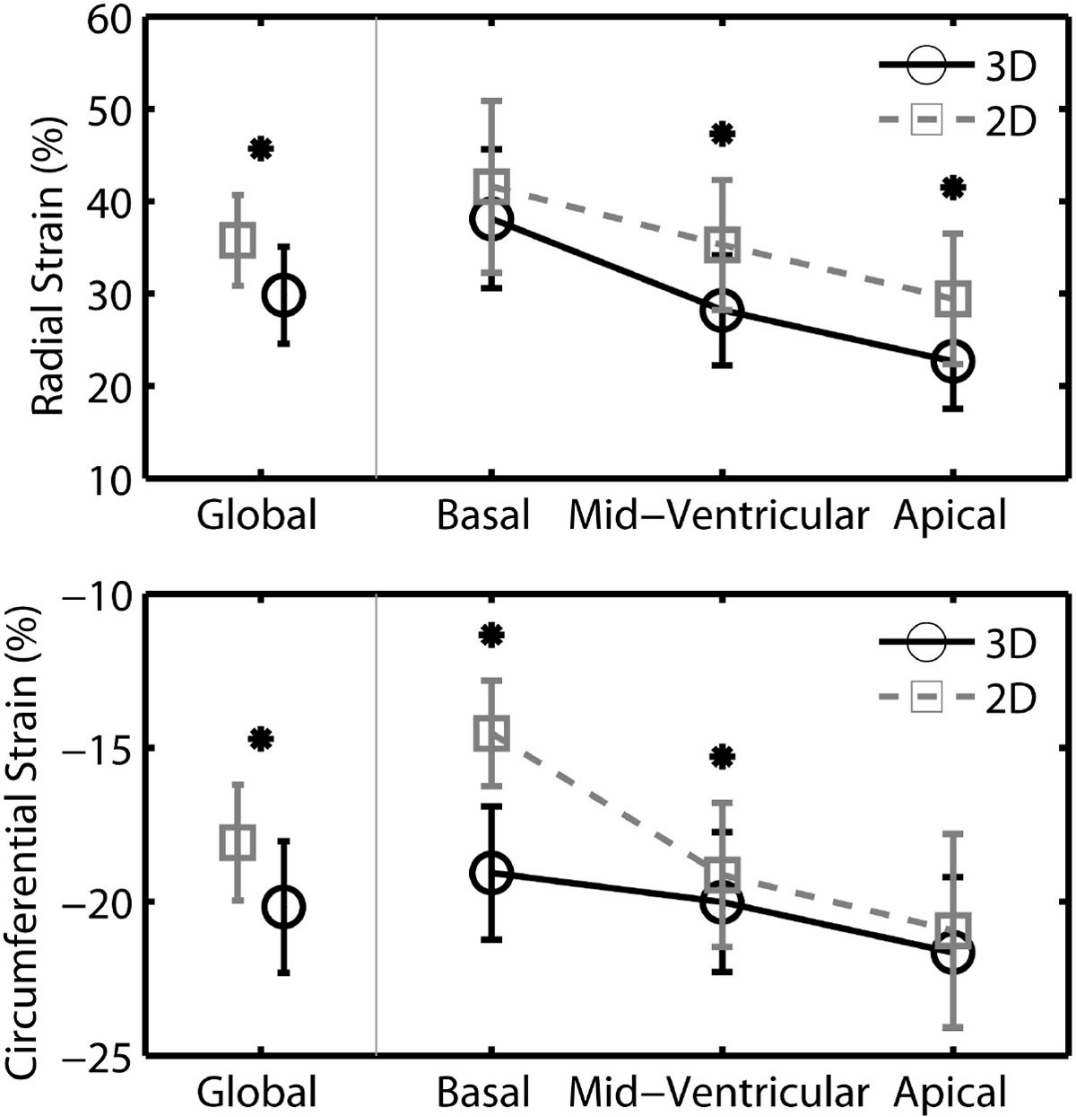
**Average (n = 8) peak radial (top) and circumferential (bottom) left ventricular strains computed with (3D) and without (2D) accounting for through-plane motion**. Bars indicate standard deviations. * denotes p < 0.05 for 3D vs 2D.

